# CD36 deletion prevents white matter injury by modulating microglia polarization through the Traf5-MAPK signal pathway

**DOI:** 10.1186/s12974-024-03143-2

**Published:** 2024-06-05

**Authors:** Xiaoxiang Hou, Xiaolin Qu, Wen Chen, Xianzheng Sang, Yichao Ye, Chengqing Wang, Yangu Guo, Hantong Shi, Chengzi Yang, Kaixin Zhu, Yelei Zhang, Haoxiang Xu, Liquan Lv, Danfeng Zhang, Lijun Hou

**Affiliations:** 1 Department of Neurosurgery, Changzheng Hospital, Naval Medical University, 415 Fengyang Rd, Shanghai, China; 2 Department of Neurosurgery, The First Affiliated Hospital of Wenzhou Medical University, Wenzhou Medical University, Wenzhou, China; 3 Department of Neurosurgery, The First Naval Hospital of Southern Theater Command, Zhanjiang, China; 4 Department of Neurosurgery, Xishan People’s Hospital of Wuxi City, Wuxi, China

**Keywords:** Traumatic brain injury, White matter injury, CD36, Microglial polarization

## Abstract

**Background:**

White matter injury (WMI) represents a significant etiological factor contributing to neurological impairment subsequent to Traumatic Brain Injury (TBI). CD36 receptors are recognized as pivotal participants in the pathogenesis of neurological disorders, including stroke and spinal cord injury. Furthermore, dynamic fluctuations in the phenotypic polarization of microglial cells have been intimately associated with the regenerative processes within the injured tissue following TBI. Nevertheless, there is a paucity of research addressing the impact of CD36 receptors on WMI and microglial polarization. This investigation aims to elucidate the functional role and mechanistic underpinnings of CD36 in modulating microglial polarization and WMI following TBI.

**Methods:**

TBI models were induced in murine subjects via controlled cortical impact (CCI). The spatiotemporal patterns of CD36 expression were examined through quantitative polymerase chain reaction (qPCR), Western blot analysis, and immunofluorescence staining. The extent of white matter injury was assessed via transmission electron microscopy, Luxol Fast Blue (LFB) staining, and immunofluorescence staining. Transcriptome sequencing was employed to dissect the molecular mechanisms underlying CD36 down-regulation and its influence on white matter damage. Microglial polarization status was ascertained using qPCR, Western blot analysis, and immunofluorescence staining. In vitro, a Transwell co-culture system was employed to investigate the impact of CD36-dependent microglial polarization on oligodendrocytes subjected to oxygen-glucose deprivation (OGD).

**Results:**

Western blot and qPCR analyses revealed that CD36 expression reached its zenith at 7 days post-TBI and remained sustained at this level thereafter. Immunofluorescence staining exhibited robust CD36 expression in astrocytes and microglia following TBI. Genetic deletion of CD36 ameliorated TBI-induced white matter injury, as evidenced by a reduced SMI-32/MBP ratio and G-ratio. Transcriptome sequencing unveiled differentially expressed genes enriched in processes linked to microglial activation, regulation of neuroinflammation, and the TNF signaling pathway. Additionally, bioinformatics analysis pinpointed the Traf5-p38 axis as a critical signaling pathway. In vivo and in vitro experiments indicated that inhibition of the CD36-Traf5-MAPK axis curtailed microglial polarization toward the pro-inflammatory phenotype. In a Transwell co-culture system, BV2 cells treated with LPS + IFN-γ exacerbated the damage of post-OGD oligodendrocytes, which could be rectified through CD36 knockdown in BV2 cells.

**Conclusions:**

This study illuminates that the suppression of CD36 mitigates WMI by constraining microglial polarization towards the pro-inflammatory phenotype through the down-regulation of the Traf5-MAPK signaling pathway. Our findings present a potential therapeutic strategy for averting neuroinflammatory responses and ensuing WMI damage resulting from TBI.

**Graphical Abstract:**

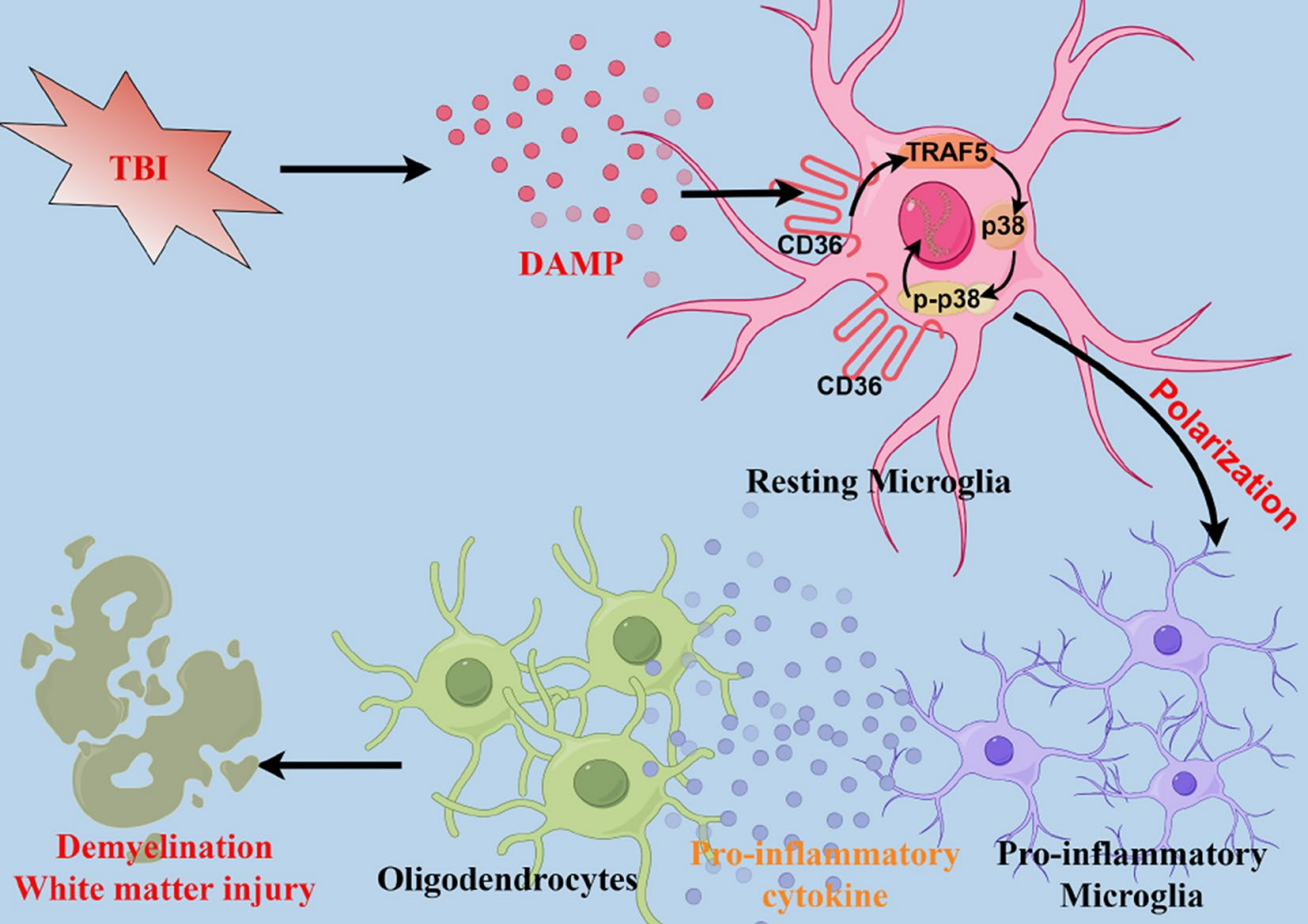

**Supplementary Information:**

The online version contains supplementary material available at 10.1186/s12974-024-03143-2.

## Introduction

Traumatic brain injury (TBI) represents a globally significant source of both mortality and morbidity [1]. It inflicts damage not only upon gray matter but also results in the occurrence of severe white matter injury (WMI) [2]. The profound white matter injury leads to substantial oligodendrocyte loss, a critical cell type responsible for myelin production, maintenance of axonal metabolism, and the regulation of neuroplasticity. This, in turn, precipitates catastrophic neurological impairment. A substantial body of preclinical research has concentrated primarily on the treatment of gray matter injury, with relatively fewer investigations into white matter injury. This underrepresentation may, in part, contribute to the suboptimal therapeutic outcomes observed.

TBI comprises two sequential phases of injury: the initial mechanical trauma to the head caused by external forces resulting in direct brain tissue damage, and the secondary biochemical cascades, including inflammatory responses and other pathophysiological complications [3]. WMI is exacerbated by potent and persistent inflammatory reactions following TBI [4]. Consequently, the management of secondary biochemical cascades, particularly inflammation, is of paramount importance for neurological recovery. Microglia, as pivotal players in the neuroinflammatory response following TBI [5], undergo a phenotypic shift from a resting state to either pro-inflammatory or anti-inflammatory phenotype upon stimulation [6, 7]. Pro-inflammatory microglia are capable of promoting the production of inflammatory factors, thereby exacerbating neurological damage [8]. In contrast, anti-inflammatory phenotype facilitates the release of pro-neural repair neurotrophic factors [9]. Pro-inflammatory microglia, induced by LPS and IFN-γ, generate substantial inflammatory factors and oxidative metabolites, while anti-inflammatory microglia, stimulated by IL-4, promote vascular regeneration [10]. In vitro experiments reveal that pro-inflammatory microglia induce apoptosis in neurons and oligodendrocyte precursor cells (OPCs), and hinder the maturation of oligodendrocytes. Conversely, anti-inflammatory microglia promote neuronal survival, synapse formation, the generation of mature oligodendrocytes, and the regeneration of myelin sheaths, thus implicating their potential role in white matter repair [11]. Therefore, the manipulation of the balance between pro- and anti-inflammatory phenotypes offers promising avenues for novel neuroprotective strategies to mitigate secondary WMI in TBI patients.

CD36, a type B scavenger receptor, is predominantly expressed on various cell types including monocytes/macrophages, dendritic cells, microglia, hepatocytes, and microvascular endothelial cells [12]. Depending on the ligand, CD36 activates a multitude of intracellular signaling pathways. While earlier research emphasized CD36’s involvement in lipid metabolism, recent studies have identified its role in the neuroinflammatory processes underlying several central nervous system (CNS) diseases such as ischemic brain injury, Alzheimer’s disease, and spinal cord injury. CD36 has been found to contribute to neuroinflammatory reactions, including oxidative stress and endoplasmic reticulum responses, through the mediation of NF-κB, MAPK, and other pathways following various CNS injuries [13–15]. Notably, the inhibition of CD36 by SSO has been demonstrated to significantly reduce the levels of inflammatory factors possibly by influencing microglial polarization, thus alleviating neurological damage [16, 17].

In light of these considerations, our study was devised to investigate the role and underlying mechanisms of CD36 in microglial polarization and WMI subsequent to TBI.

## Materials and methods

### Experimental design

To elucidate the influence and underlying mechanisms of CD36 receptors on white matter injury and neurological function in murine models post-TBI, C57BL/6J wild-type mice were randomly allocated into three cohorts: a sham group, a TBI group, and a TBI group treated with SB242235. Additionally, CD36-deficient C57BL/6J mice were randomly distributed into a sham group and a TBI group. Lentiviral constructs designed to overexpress or knockdown CD36, denoted as lenti-CD36OE-shRNA and lenti-CD36KD-shRNA respectively, as well as lenti-Traf5KD-shRNA, were engineered. BV2 microglial cells were stably transfected with one or more of the aforementioned lentiviruses. These cells, alongside non-transfected and SB242235-treated counterparts, were exposed to LPS (100ng/ml) and IFN-γ (20ng/ml) for 24 h, then categorized into distinct experimental groups. Concurrently, primary oligodendrocytes were divided into a control group and an OGD group, with the latter further subdivided based on co-culture conditions with microglia under various treatment scenarios. Randomization of animal and cell groupings was executed using a lottery-drawing box method. All primary outcome measures were conducted by researchers blinded to group allocation and experimental conditions. Animals that perished during or subsequent to the injury were omitted from the analysis. The time points of the experiments corresponding to different results are shown in Sch. 1.

**Sch. 1:**
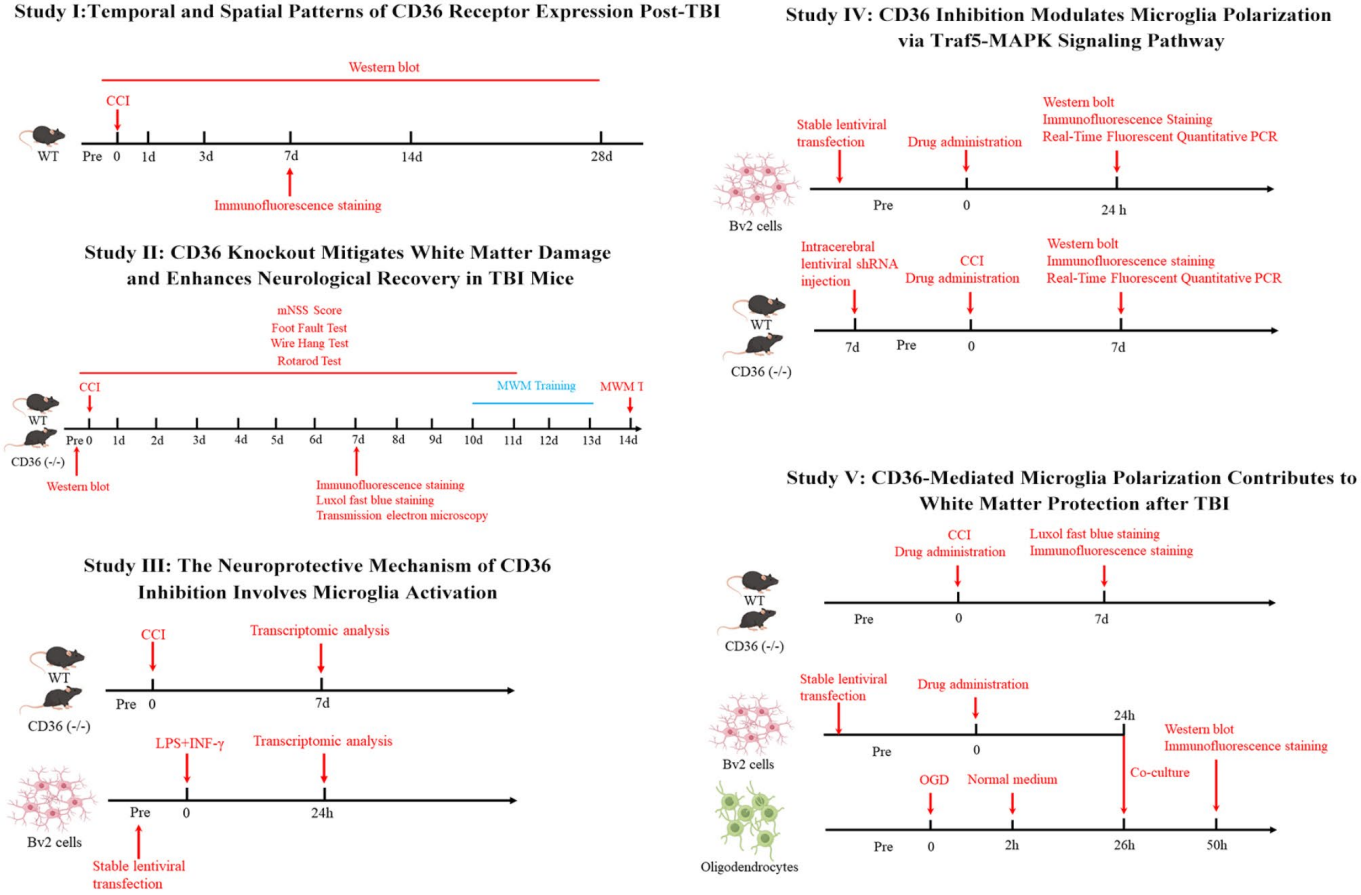
Schematic diagram of time points for experiments of this study. CCI: controlled cortical impact; OGD: oxygen-glucose deprivation

### Animals

Male C57BL/6J wild-type and CD36 knockout mice, aged 8 weeks, were procured from MODEL ORGANISMS (Shanghai, China). In total, 148 mice were utilized in our study. Of these, 107 underwent TBI procedures, with 3 succumbing to surgical complications. Consequently, data from 145 mice were incorporated into our analysis. All experimental procedures were rigorously conducted in accordance with the Guide for the Care and Use of Laboratory Animals as promulgated by the US National Institutes of Health (NIH publication No.85 − 23, revised 1996) and received approval from the Animal Care and Use Committee of the Naval Medical University (Approval ID:82,201,519). Measures were taken to minimize both the number of animals utilized and their suffering.

### TBI and sham injury

TBI was induced using a controlled cortical impact (CCI) methodology as previously described [18, 19]. Animals were randomized into experimental groups and anesthetized with 1.5% isoflurane in a 30% O_2_/68.5% N_2_O mixture, allowing for spontaneous respiration. A craniotomy was performed, and the CCI was administered 2 mm lateral to the midline and 1.0 mm anterior to the bregma using a pneumatically driven device with a 3-mm flat-tipped impounder (velocity, 3.5 m/s; duration, 150 ms; depth, 1.5 mm). Post-impact, the bone flap was repositioned and sealed. Rectal temperature was maintained at 37 °C ± 0.5 °C during and up to 30 min post-procedure using a heating pad. Sham-operated animals underwent all surgical procedures except the CCI.

### Drug administration

In this study, SB242235 was used as an inhibitor of p38 MAPK due to its characteristics as a selective inhibitor of the p38 MAPK, with minimal influence on other MAPK pathways [20, 21]. SB242235 (MedChemExpress, USA) was suspended in 0.5% methyl cellulose (MedChemExpress, USA) and administered via a single oral gavage at a dose of 100 mg/kg immediately after the mice regained consciousness post-TBI. The in vivo administration protocols for SB242235 have been detailed previously [20]. A dose-response study confirmed that optimal p-p38 inhibition was observed at a dose of 100 mg/kg for in vivo experiments and at a concentration of 5µM for in vitro experiments (Fig. S1).

### Intracerebral lentiviral shRNA infection

For intracerebral lentiviral shRNA administration, viral particles containing shRNA targeting Traf5 or a non-targeting sequence were stereotactic injected into the right corpus callosum via cranial drilling (0.75 mm lateral and 0.82 mm posterior to the bregma, 1.75 mm ventral to the skull surface) 7 days prior to TBI. The infusion was conducted at a rate of 0.1 µL/min over 50 min using a microinfusion system connected to a microsyringe pump (UMP2; World Precision Instruments). This procedure did not increase mortality rates among the animal subjects.

### Behavioral tests

#### The mNSS test

The mNSS test consists of ten different tasks that can evaluate the motor (muscle status, abnormal movement), sensory (visual, tactile and proprioceptive), balance, and reflex functions of mice. Neurological function was graded from 0 to 18 (0 = normal function; 18 = maximal deficit). One point was scored for each abnormal behavior or for the lack of a tested reflex. Therefore, higher scores implying greater neurological injury.

#### The rotarod test

Briefly, mice were forced to run on a rotating drum (IITC Life Science, Woodland Hills, CA, USA) with speeds starting at 4 rpm and accelerating to 40 rpm within 300 s. Three consecutive trials were conducted for each mouse with an interval of 15 min. The time at which a mouse fell off the drum was recorded as the latency to fall. Data were expressed as mean values from three trials.

#### Foot fault test

Mice were placed on hexagonal wire mesh grids of different sizes. As the mice moved along the wire mesh, their forepaws either stepped or slipped. The number of times these footsteps are recorded, as well as the total number of steps the mouse takes across the grid. After recording the data, the ratio of the number of forepaw steps missed to the total number of forepaw steps was calculated, with larger ratios representing greater injury.

#### Wire hang test

The wire hang test was performed as described previously [22]. The experimental apparatus was a stainless-steel bar (50 cm long, 2-mm diameter) resting on two vertical supports and elevated 37 cm above a flat surface. Mice were placed on the bar midway between the supports and were observed for 30 s in four trials. The amount of time spent hanging was recorded and scored according to the following criteria: 0, fell off; 1, hung onto the bar with two forepaws; 2, hung onto the bar with an added attempt to climb onto the bar; 3, hung onto the bar with two forepaws and one or both hind paws; 4, hung onto the bar with all four paws and with tail wrapped around the bar; 5, escaped to one of the supports.

#### Morris water maze test

Cognitive function was analyzed with the Morris water maze test 10–14 d after CCI, as described previously [23]. In the learning test, a square platform (11 × 11 cm2) was submerged 2 cm beneath the water surface in a circular pool (diameter = 109 cm) of opaque water. Mice were placed into the pool from one of the four locations and allowed to locate the hidden platform for 90 s. Each mouse was trained on 4 trials (with randomly assigned starting positions) per day to locate the platform for four consecutive days. At the end of each trial, the mouse was placed on the platform or allowed to stay on the platform for 30 s with prominent spatial cues displayed around the room. 4 trials were performed on each day. The memory test was performed on day 5. The platform was removed, and a single 60-s probe trial was conducted. Time spent and traveling distance in the goal quadrant where the platform was previously located was recorded.

### BV2 cell cultures

BV2 microglial cells, procured from the National Infrastructure of Cell Line Resource, were cultured at a density of 1–2 × 10^5 cells per well in a 6-well plate format. The culture medium consisted of Dulbecco’s Modified Eagle Medium (DMEM, Gibco, 11965092, USA) supplemented with 0.1% penicillin-streptomycin (Sigma, P4333, St. Louis, MO, USA) and 10% fetal bovine serum (Gibco, 10099141 C, USA). The cells were incubated at 37 °C in a humidified atmosphere containing 5% CO2 for 48 h prior to subsequent experimental procedures.

### Primary OPC and oligodendrocyte cultures

Primary oligodendrocyte precursor cell (OPC) cultures were derived using the differential adhesion technique. Brains from neonatal C57 mice (postnatal days 1–2) were triturated post-meningeal and vascular tissue removal. The dissociated brain tissues were treated with 0.01% Trypsin (Sigma, T4049, USA) at 37 °C for 15 min. Following trypsinization, the cells were washed with ice-cold DMEM, filtered through a 70-µm cell strainer (CORNING, USA), and seeded onto poly-D-lysine (Sigma, P6307, USA)-coated T-flasks (CORNING, USA) containing culture media (DMEM/F12 (Gibco, 11320033, USA) supplemented with 10% heat-inactivated FBS (Gibco, 10099141 C, USA), 2 mM L-glutamine (Gibco, A2916801, USA), 1 mM sodium pyruvate (Gibco, 11360070, USA), 100 µM nonessential amino acids (Gibco,11,140,050, USA), 50 U/mL penicillin (Sigma, 76427, USA), and 50 µg/mL streptomycin (Sigma, 85886, USA) [24]. The cultures were incubated at 37 °C in a 5% CO2 atmosphere for 10–12 days. Microglia were detached by shaking the flasks at 180 rpm for 1 h. After a resting period of 1 h in the incubator, the flasks were shaken overnight at 200 rpm to separate OPCs from the astrocyte layer. OPCs were cultured for 3–5 days in serum-free basal defined medium (BDM: DMEM, 0.1% bovine serum albumin (Gibco, 11021037, USA), 50 µg/mL human apo-transferrin (Sigma, 616395, USA), 50 µg/mL insulin (Sigma, I9278, USA), 30 nM sodium selenite (Sigma, S5261,USA), 10 nM D-biotin (Invitrogen, B1595, USA), 10 nM hydrocortisone (Sigma, 3867, USA) supplemented with 10 ng/mL platelet-derived growth factor (Gibco, 31517, USA) and 10 ng/mL basic fibroblast growth factor (Gibco, PMG0031, USA). For differentiation into mature oligodendrocytes, OPCs were treated with triiodothyronine (Sigma, T2877, USA) and ciliary neurotrophic factor (Sigma, 01-195, USA) at 10 ng/mL each. The culture medium was refreshed every two days. Sources of material for cell culture are listed in Table. S1.

### Co-culture of BV2 cells with oligodendrocytes

Oligodendrocytes were subjected to oxygen-glucose deprivation (OGD) for 2 h and then returned to a normoxic medium for 24 h. In a Transwell system, which permits communication via soluble factors, pre-treated BV2 cells were cultured in the upper chamber, while control or post-OGD oligodendrocytes were placed in the lower chamber for an additional 24 h.

### Transmission electron microscopy

Following perfusion with ice-cold saline and fixatives (4% paraformaldehyde and 2.5% glutaraldehyde in 0.1 M phosphate-buffered saline), corpus callosum (CC) tissue proximal to the injury site was excised into 1 mm^3 blocks. These samples were post-fixed in 2% glutaraldehyde for 24 h, washed in PBS, and further fixed in 1% osmium tetroxide in 0.1 M PBS for 45 min. The tissue was dehydrated in a graded series of acetone and embedded in Araldite resin. Ultrathin Sect. (60 nm) were prepared using a Leica UCT ultramicrotome with a diamond knife (Diatome, Wetzlar, Germany), stained with uranyl acetate and lead citrate, and examined with a JEM1400 transmission electron microscope (JEOL, Akishima, Japan). Each dissected 1 mm^3 blocks tissue block was bisected, and one section from each half was processed for electron microscopy. Subsequently, two sections from each animal were analyzed, capturing 3–5 images (600 μm^2 each) at random within the CC at 50,000× magnification. At least 200 axons were analyzed per animal. Images were analyzed using ImageJ software by an investigator blinded to the experimental groups. G-ratios were calculated as the ratio of the inner axonal diameter to the total outer diameter (axonal diameter plus myelin sheath thickness).

### Luxol fast blue staining

Coronal brain sections of 25 μm thickness were subjected to Luxol Fast Blue staining as per the protocol provided by American Mastertech to assess myelin integrity. Sections were incubated for 1 h in a preheated Luxol Fast Blue solution at 60 °C. Subsequently, sections were rinsed with water and differentiated in a lithium carbonate solution for 30 s, followed by a 30-second immersion in 70% ethanol. After rinsing, differentiation was assessed microscopically. The sections were then dehydrated with alcohol and coverslipped for microscopic evaluation.

### Western blot analysis

Protein extracts were obtained from the corpus callosum of mice, BV2 cells, or primary oligodendrocytes. Protein concentrations were quantified using the Bio-Rad Protein Assay with bovine serum albumin as the standard. Fifty micrograms of protein per sample were resolved by SDS-PAGE and transferred onto PVDF membranes. These membranes were incubated with primary antibodies at 4 °C overnight, followed by incubation with secondary antibodies (1:2000, AS014, Abclonal). Primary antibodies used included Anti-CD36 (1:1000, GB112562-100, Servicebio), Anti-iNOS (1:1000, ab178945, Abcam), Anti-Arg1 (1:1000, A4923, Abclonal), Anti-p38 (1:1000, 8690, CST), Anti-phospho-p38 (1:1000, 4511, CST), Anti-Traf5 (1:1000, 12868-1-AP, Proteintech), and Anti-MBP (1:1000, A11162, Abclonal). β-actin (1:100000, AC026, Abclonal) served as the loading control. Blots were visualized using a BIO-RAD imaging system, and band intensities were quantified using ImageJ software, normalized to β-actin.

### Real-time fluorescent quantitative PCR (RT-qPCR)

Total RNA was isolated using an RNA-extraction kit (Fastagen, China) following the manufacturer’s instructions and reverse-transcribed into cDNA (321,392, Promega, Beijing, China). The cDNA was combined with gene-specific primers and SYBR Premix Ex Taq™ (Takara, Japan) for qPCR quantification using an Applied Biosystems instrument. Primer sequences are detailed in Table 1. The relative expression levels of target mRNAs were calculated using the 2–ΔΔCt method, with normalization to the housekeeping gene β-actin.

**Table 1:** qPCR primer sequences

Gene	Forward primer (5′-3′)	Reverse primer (5′-3′)
CD36	CATTTGCAGGTCTATCTAC	CAATGTCTAGCACACCATAAG
iNOS	CAAGCACCTTGGAAGAGGAG	AAGGCCAAACACAGCATACC
Arg1	TCACCTGAGCTTTGATGTCG	CTGAAAGGAGCCCTGTCTTG
β-actin	GCTCTCCTATGTTGCTCTAG	CGCTCCTTGCCAATACTC

### Immunofluorescence staining

Brain sections were permeabilized with 0.4% Triton X-100 for 10 min, followed by three 5-minute PBS washes. Sections were then blocked with 2% bovine serum albumin for 35 min and incubated overnight at 4 °C with primary antibodies including anti-MBP (1:200, Abclonal), anti-SMI-32(1:200, SMI-32P, BioLegend), anti-iNOS (1:400, ab178945, Abcam), anti-Arg1 (1:200, A4923, Abclonal), Anti-CD36 (1:500, GB112562-100, Servicebio), anti-GFAP (1:500, ab279289, Abcam), anti-Olig2 (1:200, ab109186, Abcam), anti-NeuN (1:100, ab104224, Abcam), and anti-Iba-1 (1:100, ab283319, Abcam). After washing with PBS, sections were incubated with appropriate secondary antibodies (secondary antibodies included the following: Alexa Fluor 488 secondary antibodies or Alexa Fluor 594 secondary antibodies (1:400, Jackson ImmunoResearch) for 2 h at room temperature and counterstained with DAPI for 6 min. Cultured cells were fixed with 4% PFA for 30 min, blocked, and incubated with primary antibodies overnight at 4 °C. Following secondary antibody incubation, cells were counterstained with DAPI and analyzed using ImagePro Plus 6.0.

For identifying pro-inflammatory and anti-inflammatory microglia, double-positive cells (iNOS/iba1 for pro-inflammatory microglia and ARG1/iba1 for anti-inflammatory microglia) were identified as co-localized cells and were counted using image software [25]. In Sholl analysis, 200 suitable oligodendrocytes were selected for evaluation in each independent experiment per group. For the cell count, a consistent area size was uniformly selected for cell counting in each group, ensuring a minimum count of 500 cells per independent experiment.

### Cell viability and death assays

Cell viability was assessed using the CCK-8 assay. Oligodendrocytes in 96-well plates were incubated with 20 µl of CCK-8 reagent at 37 °C in a 5% CO2 incubator for 2 h. After color development, absorbance at 450 nm was measured using a microplate reader (Elx800; BioTek Instruments).

Cell death was evaluated using the LDH assay, which measures LDH release into the culture medium indicative of membrane integrity loss. Aliquots of 100 µL from the culture medium were mixed with 150 µL of LDH reagent (Sigma, USA). The absorbance of the reaction was measured spectrophotometrically by monitoring the reduction of NAD + at 340 nm at 25 °C over 5 min. Data were expressed as a percentage of maximum LDH activity from control wells where all cells were lysed with Triton.

### mRNA high throughput sequencing and analysis

mRNA high throughput sequencing was performed by NewCore Biotech. (Shanghai, China). Briefly, total RNA was extracted using Trizol Reagent (ThermoFisher, 15,596,026). The mRNAs were purified using a NEBNext Poly(A) mRNA Magnetic Isolation Module Kit (NEB, E7490) according to the user manual. mRNA libraries were constructed by using Illumina TrueSeq mRNA sample preparation kit (Cat. No. RS-122-2101) according to the manufacturer’s instructions. Library sequencing was performed on an Illumina NovaSeq 6000 instrument with 151 bp paired end reads.

Bioinformatics data was analyzed. Briefly, fastp software (v0.20.0) was used to trim adaptor and remove low quality reads to get high quality clean reads. STAR software (v2.7.9a) was used to align the high-quality clean reads to the human reference genome (hg38). featureCounts software (v2.0) was used to get the raw gene level mRNA read counts as the mRNA expression profile. DESeq2 software (v1.30.1) was used to normalize and calculate the fold change and Pvalue between two groups. Ensembl GTF gene annotation database (v104) was used to annotate the mRNA. Gene Ontology and KEGG pathway enrichment analysis were performed with clusterProfiler R package (v3.18.1) based on the differentially expressed mRNAs. rMATS software (v4.1.1) was used to predict the alternative splicing events between two groups.

### Construction of plasmids and development of shRNA transfected BV2 cells

CD36 shRNA plasmids, CD36 plasmids Traf5 shRNA plasmids, and negative control plasmids were constructed by Genomeditech (Shanghai, China) (Table 2). The corresponding vector was pGMLV-hU6-MCS-CMV-Puromycin. BV2 cells were stably transfected by the lentiviruses (Genomeditech, Shanghai, China) following the manufacturer’s protocol. The positive clones were screened by puromycin.

**Table 2:** Target sequences

Gene	TargetSeq (5′-3′)
CD36 Knock-down #1	GCTATTGCGACATGATTAATG
CD36 Knock-down #2	GCCTGTGTATATTTCGCTTCC
Traf5 Knock-down #1	GCGGCAAGAAGAACCATATTG
Traf5 Knock-down #2	GCACCTGTCCCTGTACTTTGT

### Statistical analysis

Results were presented as mean ± standard error of the mean (SEM). GraphPad Prism software (version 8.1.0, La Jolla, CA, USA) was used for statistical analyses. The distribution normality and variance equality of the data were assessed using the Shapiro-Wilk and Levene’s variance equality tests, respectively. If the data adhered to normal distribution and the *P* value of Levene variance equality test was greater than 0.05, Student’s t-test was used for two-group comparisons and one-way ANOVA with Tukey’s post hoc test for multiple groups. Otherwise, non-parametric tests such as the Mann-Whitney U rank sum test or the Kruskal-Wallis rank sum test were employed. Differences were considered significant at *P* < 0.05.

## Results

### Temporal and spatial patterns of CD36 receptor expression post-TBI

**Fig. 1:**
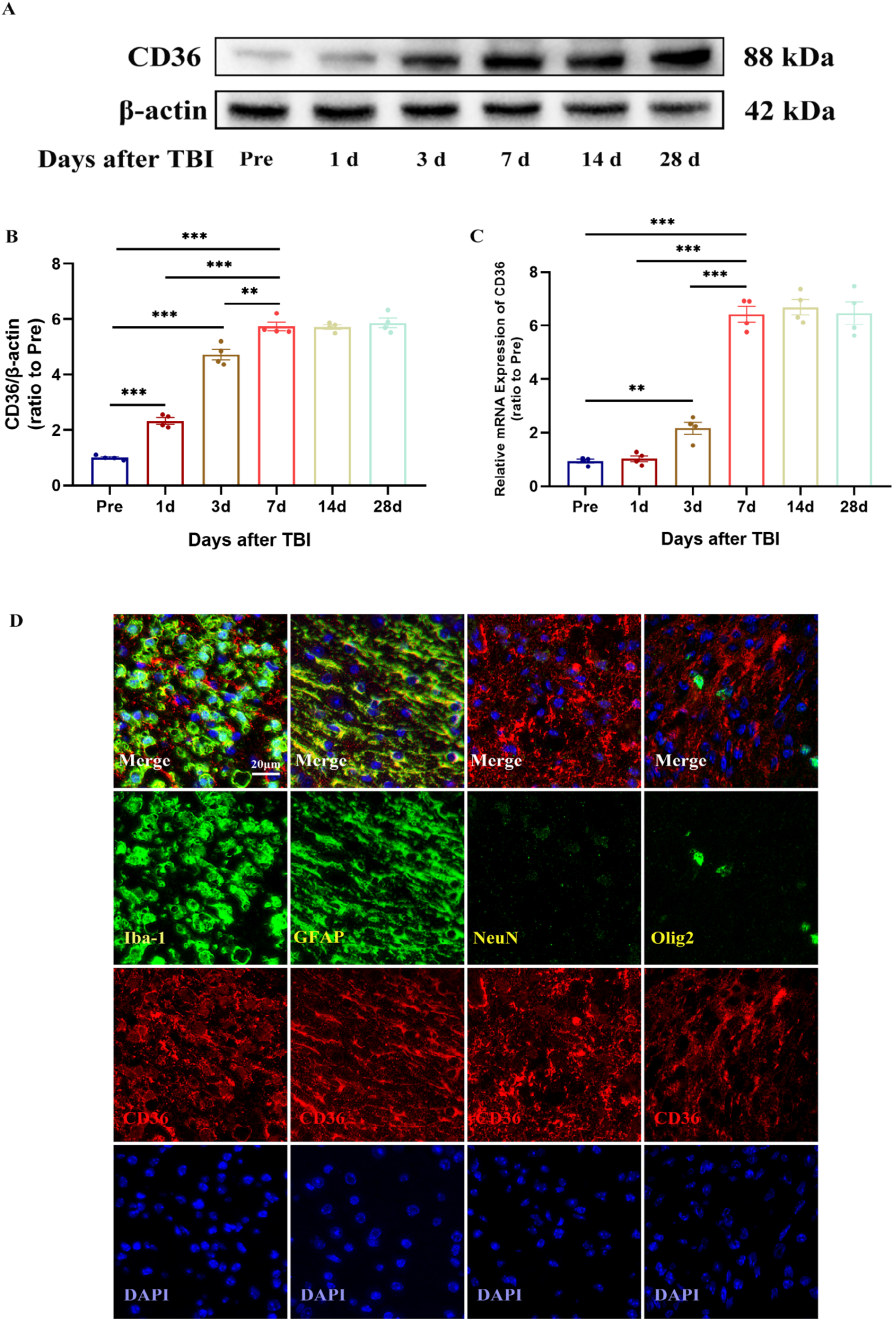
Temporal and spatial expression patterns of CD36 receptor expression after TBI. (**A**, **B**) Western blot analysis and quantification depicting the temporal expression of CD36 receptors in brain tissue post-TBI. (**C**) Quantitative PCR results showing CD36 expression trends following TBI. (**D**) Immunofluorescence images illustrating CD36 localization in neurons, microglia, astrocytes, and oligodendrocytes at day 7 post-injury within the injury and peri-injury zones. Scale bar: 20 μm. Data represent mean ± SEM for 4 mice per group. *P < 0.05, **P < 0.01, ***P < 0.001

The temporal and spatial expression patterns of CD36 receptors in the brain were examined by collecting corpus callosum tissues from control mice and those subjected to TBI at multiple time points: 1, 3, 7, 14, and 28 days post-injury. Western blot (WB) and quantitative PCR (qPCR) analyses were employed to detect CD36 expression in the peri-injury zone of brain tissue. The results indicated a progressive increase in CD36 receptor expression post-TBI, reaching a peak at day 7, as demonstrated by immunoblotting (Fig. 1A, B) and corroborated by qPCR findings (Fig. 1C). Based on these findings, day 7 post-injury was selected as the optimal time point for further observation. Immunofluorescence staining on day 7 post-injury revealed that CD36 was predominantly expressed in astrocytes (GFAP^+^) and microglia (Iba-1^+^), with minimal expression in oligodendrocytes (Olig2^+^) and neurons (NeuN^+^) within the corpus callosum (Fig. 1D).

### CD36 knockout mitigates white matter damage and enhances neurological recovery in TBI mice

**Fig. 2:**
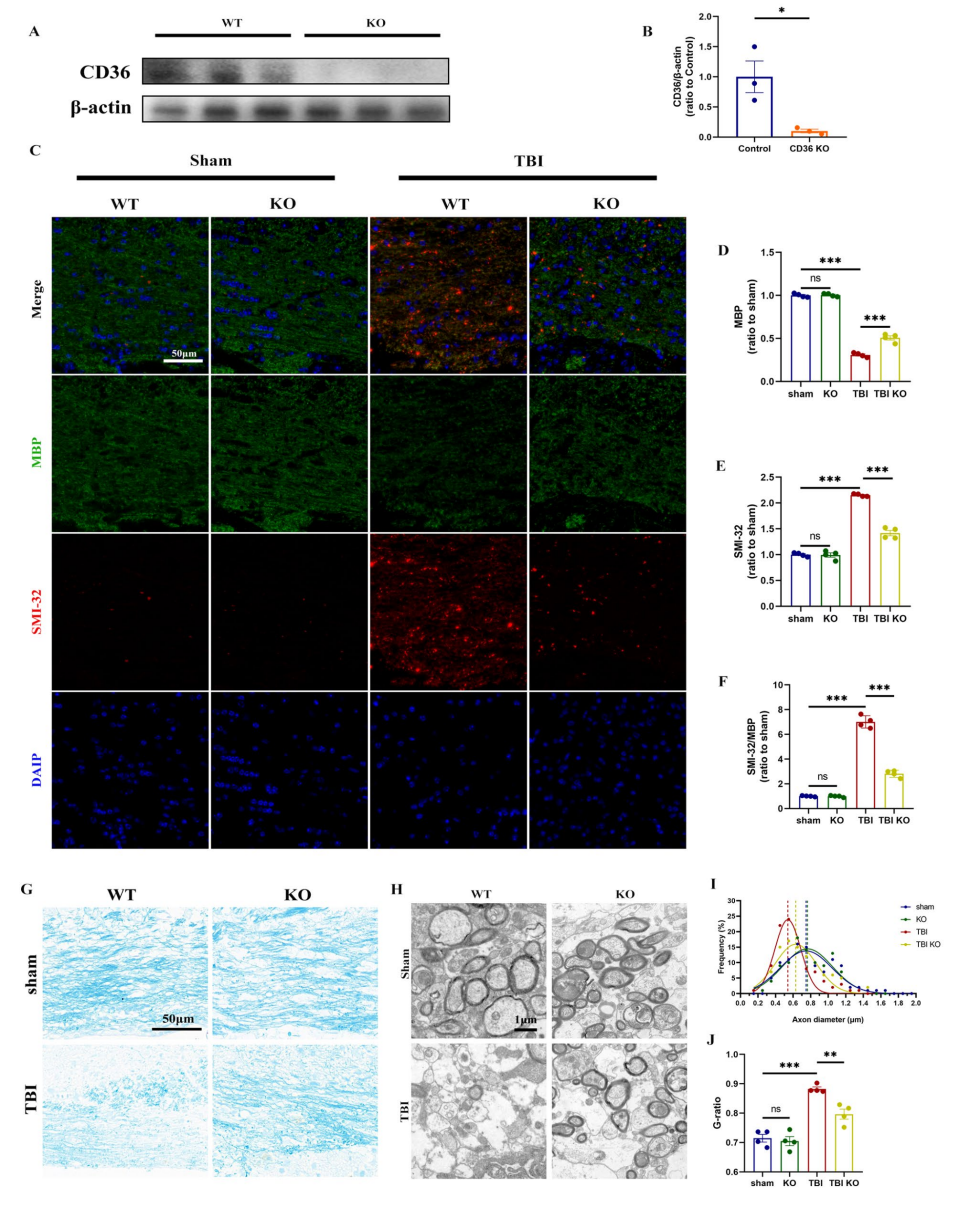
CD36 knockout mitigates white matter injury. (**A**, **B**) Validation of CD36 knockout efficiency via western blot and associated quantification, *n* = 3/group. (**C**) Luxol fast blue staining demonstrating reduced myelin loss in the corpus callosum of CD36 knockout mice at day 7 post-TBI. Scale bar: 50 μm. (**D**) Transmission electron microscopy images showing demyelination in the corpus callosum at day 7 post-TBI. Scale bar: 1 μm. (**E**) Axonal diameter distribution. (**F**) G-ratio analysis reflecting myelin thickness relative to axonal diameter. (**G**) Dual immunofluorescence staining for dephosphorylated neurofilament protein (SMI-32, red) and myelin basic protein (MBP, green) in the corpus callosum at day 7 post-TBI, with DAPI staining nuclei (blue). Scale bar: 50 μm. (**H**–**J**) Quantitative analysis of white matter injury, indicated by changes in MBP (**H**), SMI-32 (**I**), and their ratio (**J**). Data represent mean ± SEM for 4 mice per group. *P < 0.05, **P < 0.01, ***P < 0.001

The deletion of CD36 in mice was confirmed (Fig. 2A, B; Fig. S2). To evaluate whether CD36 inhibition could mitigate white matter damage post-TBI, myelin loss in the corpus callosum (CC) was assessed using Luxol fast blue staining, and alterations in myelin basic protein (MBP) and abnormally dephosphorylated neurofilament protein (detected with SMI-32 antibody) were examined. In wild-type (WT) mice subjected to controlled cortical impact (CCI), a significant loss of MBP and an increase in abnormally dephosphorylated neurofilament protein were observed. However, the ratio of SMI-32 to MBP was significantly reduced in the TBI KO group compared to the TBI WT group (Fig. 2C–F), and similar results were obtained from immunofluorescence detection of the ipsilateral striatum (Fig. S3). Luxol fast blue staining also indicated partial restoration of myelin in the CD36 KO group after TBI (Fig. 2G). Additionally, electron microscopy of the CC post-injury revealed a substantial reduction in axon diameters in the TBI group compared to both the TBI KO and sham groups (Fig. 2H, I). G-ratio analysis further confirmed a significant decrease in myelin thickness in the TBI group relative to the sham and TBI KO groups (Fig. 2J).

**Fig. 3:**
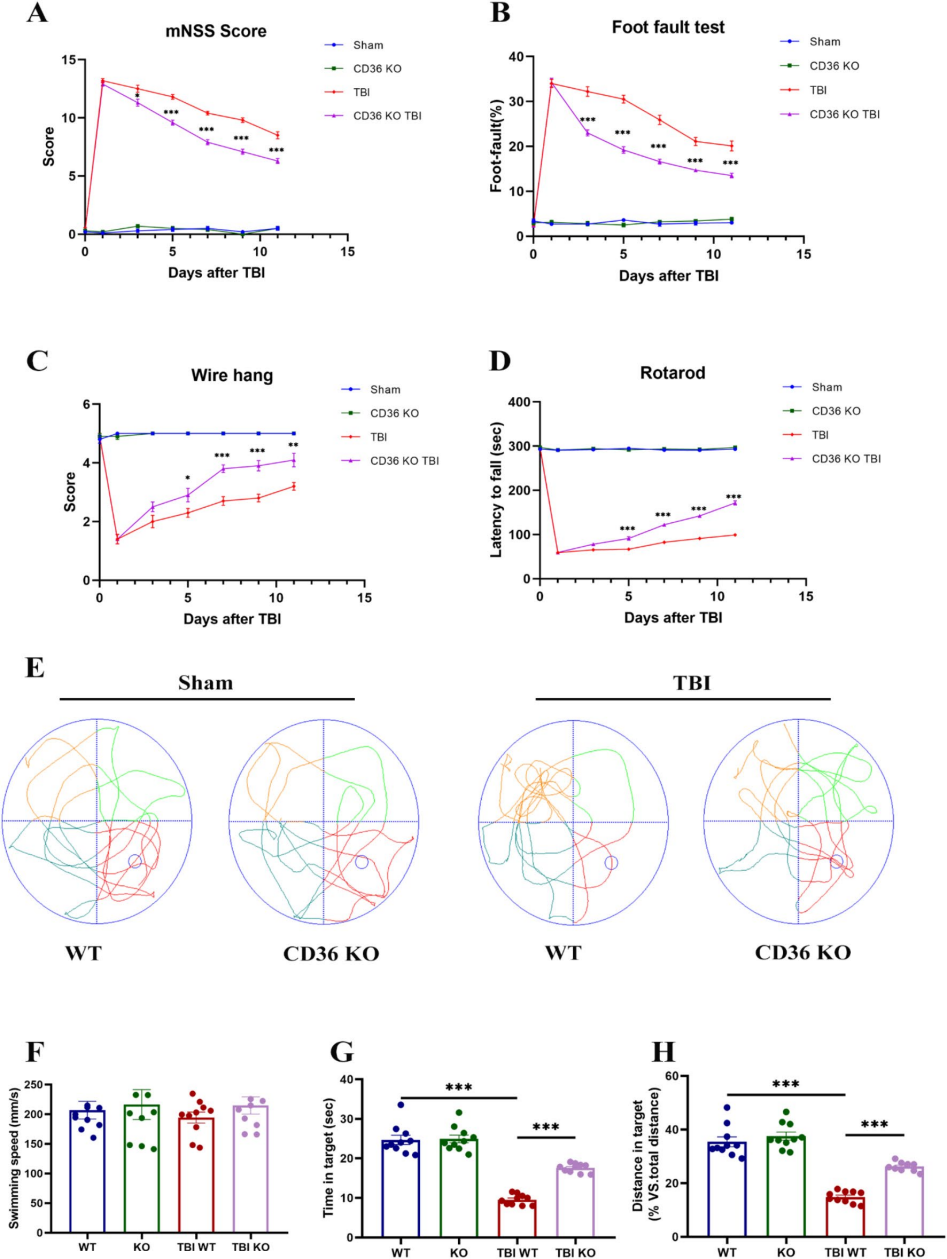
Neurological function enhancement in CD36 knockout mice. (**A**) Modified Neurological Severity Scores (mNSS) over time post-TBI. (**B**–**D**) Sensorimotor assessments including the foot fault, wire hang, and rotarod tests. (**E**–**H**) Cognitive function evaluation in the Morris water maze, with swim paths depicted during the probe trial (**E**), distance traveled (**H**), and time spent in the target quadrant (**G**), without differences in swim speed (**F**). Data represent mean ± SEM for 10 mice per group. *P < 0.05, **P < 0.01, ***P < 0.001

Neurological function was evaluated by investigators blinded to group assignments. The modified Neurological Severity Score (mNSS) was used to gauge neurological impairment, with higher scores indicating greater impairment. Post-TBI, mNSS scores surged and then gradually declined (Fig. 3A). Notably, the decline in mNSS scores was more pronounced in the TBI KO group than in the TBI WT group. Sensorimotor functions were assessed using the foot fault test, wire hang, and rotarod tests (Fig. 3B–D), which revealed that CD36 knockout mitigated sensorimotor deficits. Moreover, CD36 KO mice demonstrated cognitive improvements in the Morris water maze test (Fig. 3E), evidenced by a decrease in time spent on escape during the training phase (Fig. S4A and B) and an increase in swimming distance and time in the target quadrant after platform removal (Fig. 3G, H). No differences in swimming speed were observed among the groups during both the training and testing phases, suggesting comparable gross motor abilities (Fig. 3F, Fig. S4C).

### The neuroprotective mechanism of CD36 inhibition involves microglia activation

**Fig. 4:**
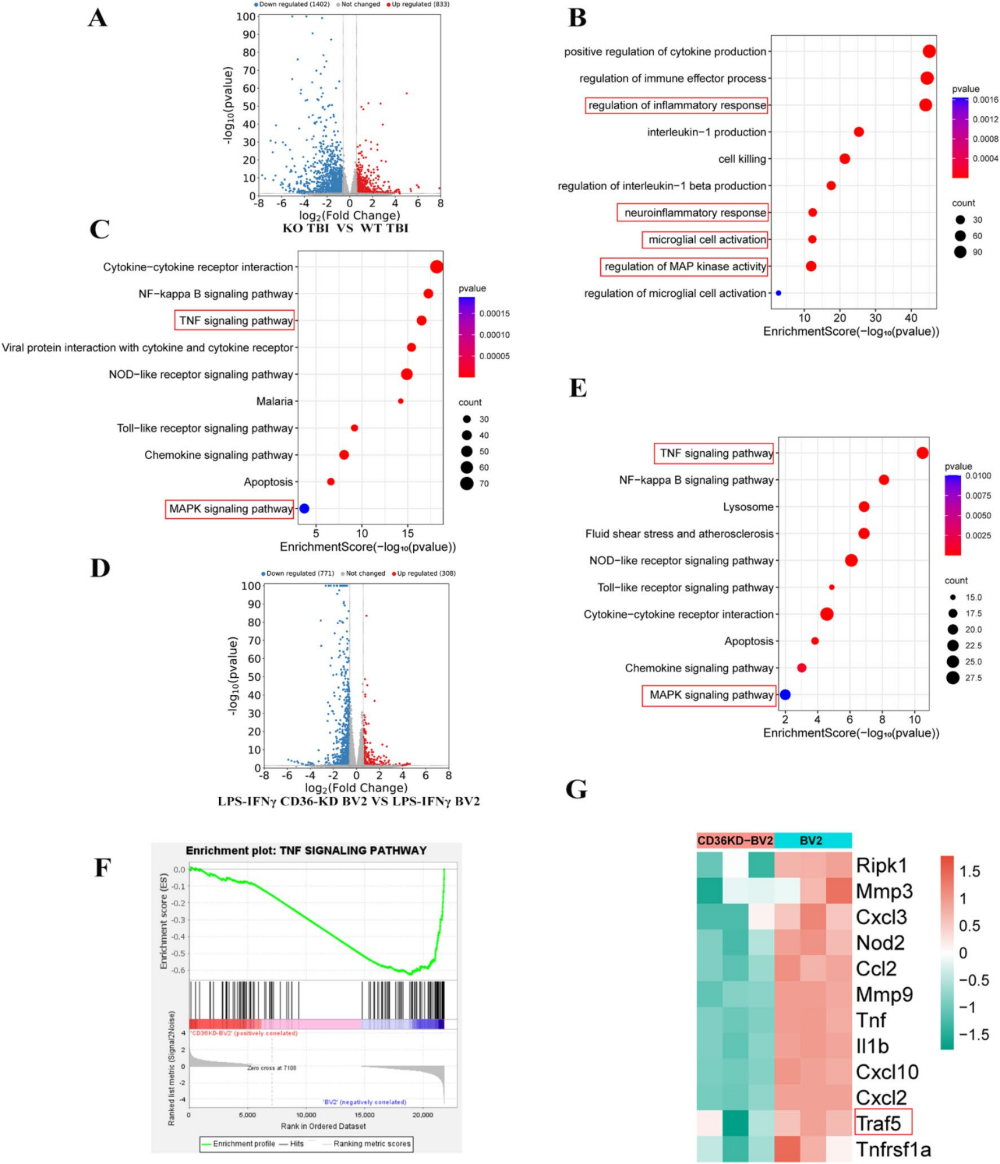
Transcriptomic analysis post-CD36 inhibition. (**A**) Volcano plot illustrating differentially expressed genes (DEGs) between CD36^(-/-)^ and wild-type mice post-TBI. (**B**) Gene Ontology (GO) enrichment analysis highlighting biological processes among DEGs. (**C**) Kyoto Encyclopedia of Genes and Genomes (KEGG) pathway enrichment analysis of DEGs. (**D**) Volcano plot of DEGs between CD36-knockdown (KD) BV2 cells and control BV2 cells treated with LPS + IFN-γ. (**E**) KEGG pathway enrichment analysis for DEGs in CD36-KD BV2 cells. (**F**) Gene Set Enrichment Analysis (GSEA) for the TNF signaling pathway. (**G**) Identification of core genes via GSEA.

To understand how CD36 inhibition exerts its neuroprotective effects after traumatic brain injury (TBI), a transcriptome analysis was performed on brain tissue collected from the peri-injury area of mice 7 days after controlled cortical impact (CCI). This analysis identified 1212 differentially expressed genes (DEGs), with 334 upregulated and 878 downregulated genes in the CD36 knockout (KO) group compared to the wild-type (WT) group after CCI injury (Fig. 4A). Gene Ontology (GO) enrichment analysis showed that these DEGs were involved in biological processes related to cytokine production, inflammatory response regulation, neuroinflammatory response, microglia activation, and interleukin production (Fig. 4B). Additionally, KEGG enrichment analysis revealed their involvement in various signaling pathways, including cytokine-cytokine receptor interaction, TNF signaling, NF-kappa B signaling, and MAPK signaling (Fig. 4C). This suggested that the neuroprotective effects of CD36 deletion might be linked to microglia activation and related inflammatory signaling pathways.

To delve deeper into the mechanism of CD36 in microglial activation, BV2 and CD36 knockdown (KD) BV2 cells were treated with LPS (lipopolysaccharide) and IFN-γ for 24 h, and their transcriptomes were sequenced. A total of 1079 DEGs were identified, with 308 upregulated and 771 downregulated genes in the CD36-KD BV2 group compared to the BV2 group (Fig. 4D). KEGG enrichment analysis of these DEGs revealed their involvement in signaling pathways like TNF signaling, NF-kappa B signaling, and MAPK signaling (Fig. 4E). Gene Set Enrichment Analysis (GSEA) indicated that genes were significantly enriched in the TNF signaling pathway after CD36 knockdown (Fig. 4F), and the core gene Traf5 was determined (Fig. 4G). Interestingly, KEGG enrichment analysis also showed that Traf5 and phosphorylated p38 (p-p38) in the MAPK signaling pathway were notably downregulated (Fig. S5).

### CD36 inhibition modulates microglia polarization via Traf5-MAPK signaling pathway

**Fig. 5:**
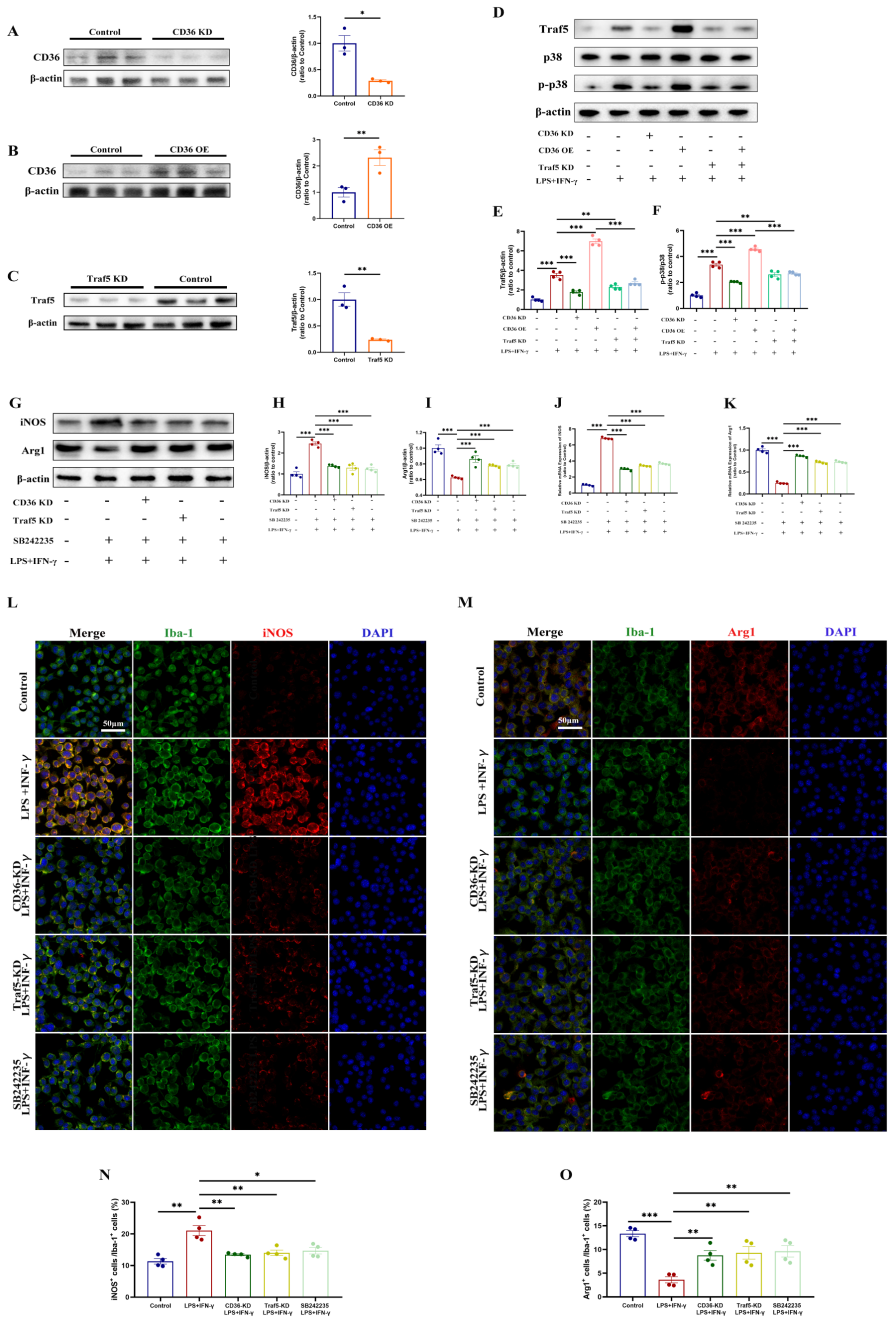
CD36 knockdown reduces pro-inflammatory polarization in BV2 cells via the Traf5-MAPK axis. (**A**–**C**) Validation of stable transfection in BV2 cells by western blot and quantification, *n* = 3/group. (**D**–**F**) Assessment of the Traf5-MAPK signaling pathway by western blot and quantification. (**G**–**I**) Western blot analysis of iNOS and ARG1 expression. (**J**, **K**) qPCR validation of iNOS and ARG1 expression. (**L**, **M**) Immunofluorescence images depicting pro-inflammatory (**L**) and anti-inflammatory (**M**) polarization in BV2 cells across different experimental conditions. (**N**, **O**) Proportional analysis of polarized BV2 cells relative to the total BV2 population. Pro-inflammatory phenotype/Total (**N**); Anti-inflammatory phenotype/Total (**O**). Data represent mean ± SEM from 4 independent experiments. *P < 0.05, **P < 0.01, ***P < 0.001

To confirm the conclusions drawn from sequencing, BV2 cells were modified with overexpression and knockdown plasmids for CD36 and Traf5. Western blot analysis demonstrated the efficiency of overexpression and knockdown (Fig. 5A–C). The effects of CD36 inhibition or overexpression on the Traf5/MAPK axis in LPS-IFN-γ-treated microglia were evaluated. LPS-IFN-γ treatment led to the upregulation of Traf5 and p-p38, and these effects were attenuated by CD36 knockdown and enhanced by CD36 overexpression. Knockdown of Traf5, either alone or in combination with CD36 overexpression, reduced p-p38 levels induced by LPS-IFN-γ (Fig. 5D–F).

**Fig. 6:**
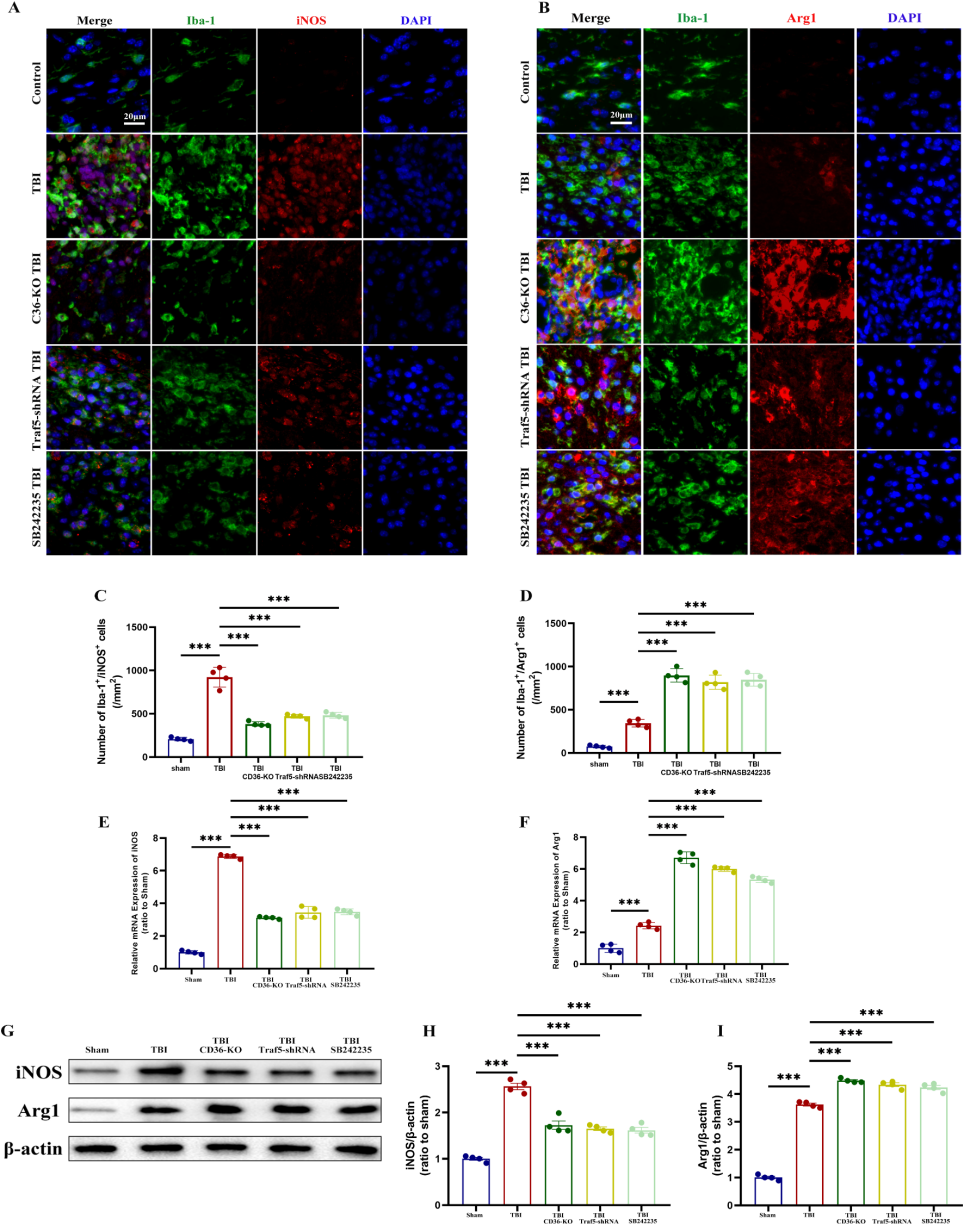
The CD36-Traf5-MAPK axis influences microglial polarization post-TBI. (**A**, **B**) Immunofluorescence images of pro-inflammatory (**A**) and anti-inflammatory (**B**) microglia polarization in the corpus callosum following TBI in different experimental groups. (**C**, **D**) Quantification of Iba1^+^/iNOS^+^ double-positive cells (**C**) and Iba1^+^/Arg1^+^ double-positive cells (**D**). (**E**, **F**) mRNA expression levels of iNOS (**E**) and Arg1 (**F**) in the corpus callosum at 7 days post-TBI. (**G**–**I**) Western blot analysis and quantification of iNOS and ARG1 expression. Data represent mean ± SEM for 4 mice per group. *P < 0.05, **P < 0.01, ***P < 0.001

The role of the CD36-Traf5-MAPK axis in microglia polarization was further confirmed through Western blot analysis, qPCR, and immunofluorescence staining. Pro-inflammatory microglia were characterized by high iNOS expression, while anti-inflammatory microglia were defined by high Arg1 expression. LPS-IFN-γ treatment induced pro-inflammatory polarization, as evidenced by elevated iNOS expression, but CD36 and Traf5 knockdown, as well as p38 inhibition, mitigated this effect. On the other hand, LPS-IFN-γ inhibited anti-inflammatory polarization, demonstrated by a decreased Arg1/Iba-1 ratio, and this effect was reversed by CD36 knockdown, Traf5 knockdown, and p38 inhibition (Fig. 5G). Immunofluorescence staining also supported these findings. Moreover, in vivo experiments conducted 7 days post-TBI showed increased density of pro-inflammatory (Iba1^+^/iNOS^+^ co-localized cells) and anti-inflammatory (Iba1^+^/Arg1^+^ co-localized cells) microglia in the injury area. In line with the in vitro results, CD36, Traf5 knockdown, and p38 inhibition promoted microglia polarization toward anti-inflammatory phenotype and decreased polarization toward pro-inflammatory phenotype (Fig. 6A–D). Additionally, Western blot and qPCR analysis confirmed the increased expression of both iNOS and Arg1 post-TBI, and CD36 inhibition, Traf5 knockdown, and p38 inhibition attenuated this effect (Fig. 6E–I).

### CD36-mediated microglia polarization contributes to white matter protection after TBI

**Fig. 7:**
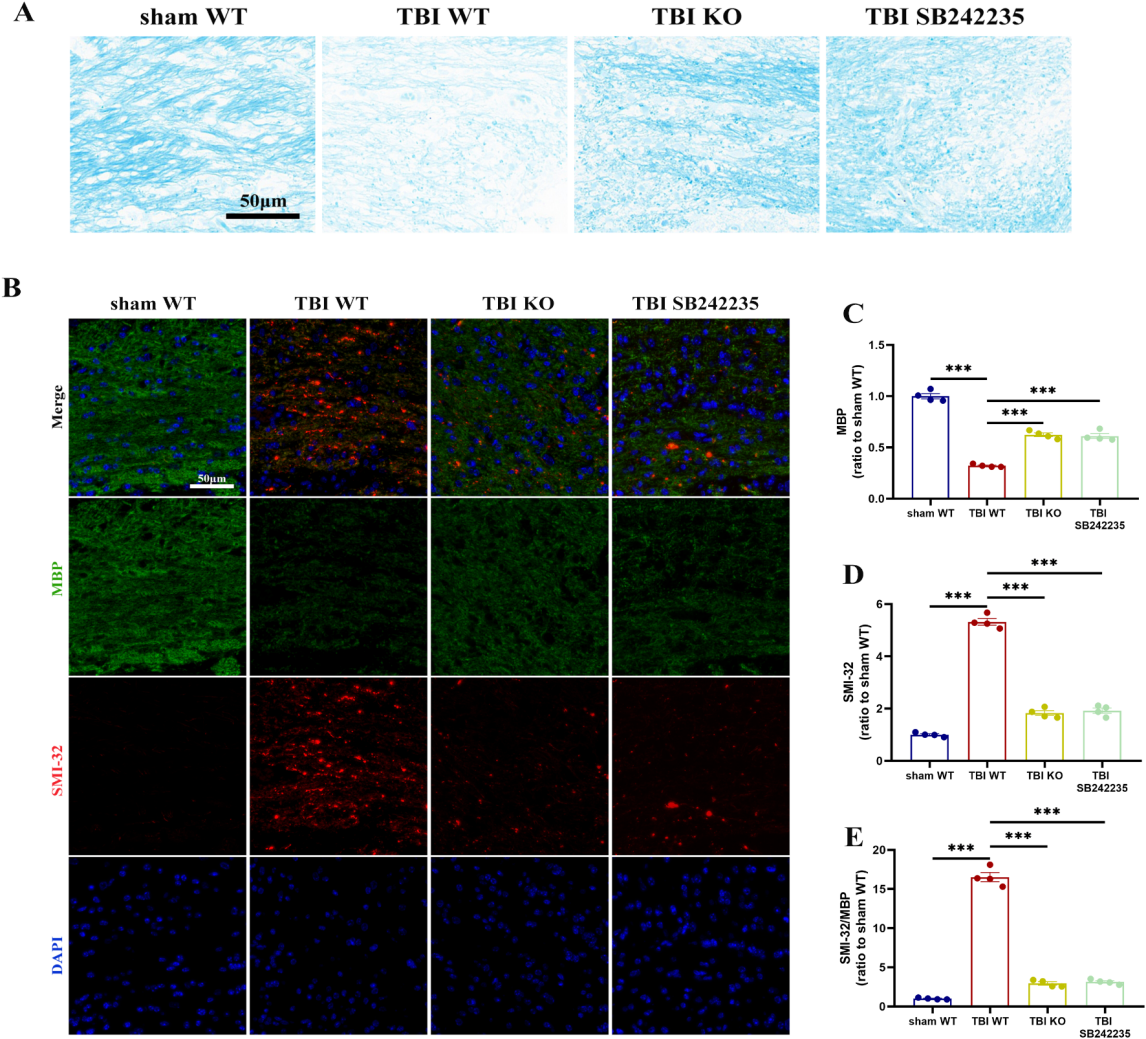
CD36-MAPK pathway inhibition reduces white matter injury associated with TBI. (**A**) Luxol Fast Blue (LFB) staining in the corpus callosum at day 7 post-TBI across different groups. Scale bar: 50 μm. (**B**) Dual immunofluorescence staining for SMI-32 (red) and MBP (green) in the corpus callosum at day 7 post-TBI, with DAPI-stained nuclei (blue). Scale bar: 50 μm. (**C**–**E**) Quantitative assessment of white matter injury, indicated by changes in MBP (**C**), SMI-32 (**D**), and their ratio (**E**). Data represent mean ± SEM for 4 mice per group. *P < 0.05, **P < 0.01, ***P < 0.001

The impact of CD36-mediated microglia polarization on white matter injury was further validated. Luxol fast blue (LFB) staining revealed significant myelin loss after TBI, which was attenuated by CD36 knockout or SB242235 treatment (Fig. 7A). This finding was consistent with the SMI-32/MBP ratio, which increased after TBI but was reversed by CD36 knockdown and SB242235 (Fig. 7B–E).

**Fig. 8:**
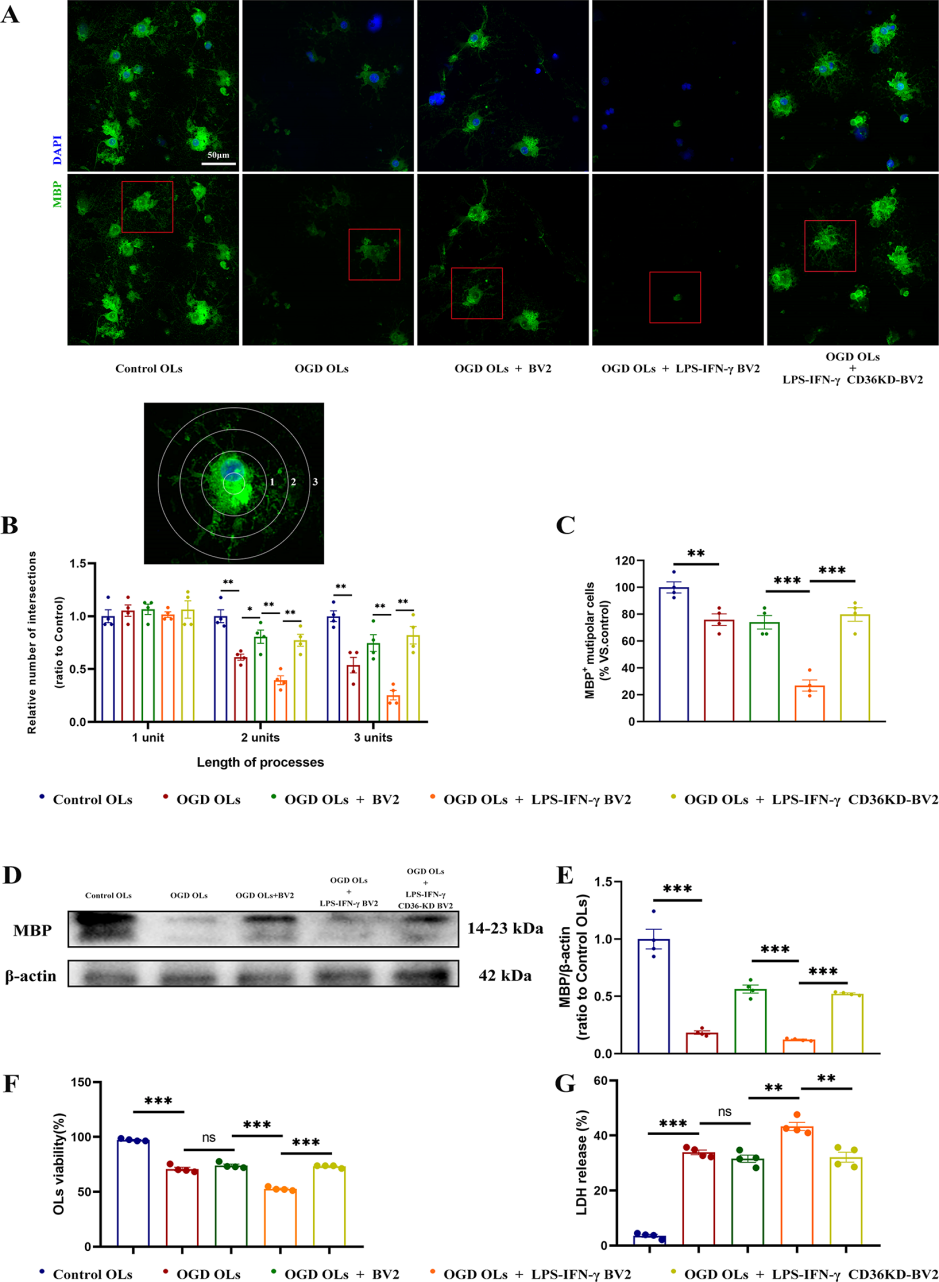
Modulation of oligodendrocyte damage by CD36-MAPK axis inhibition in a BV2 cell co-culture system under oxygen-glucose deprivation (OGD). (**A**) Immunofluorescence staining of primary oligodendrocytes in various experimental conditions. Scale bar: 50 μm. (**B**) Sholl analysis of MBP ^+^ oligodendrocyte process branching. (**C**) Count of MBP ^+^ multipolar oligodendrocytes. (**D**, **E**) Western blot validation of MBP expression levels. (**F**, **G**) Oligodendrocyte viability and cell death assessed by CCK-8 assay (**F**) and lactate dehydrogenase (LDH) release (**G**), respectively. Data represent mean ± SEM from 4 independent experiments. *P < 0.05, **P < 0.01, ***P < 0.001

To investigate the role of CD36-regulated microglia polarization in oligodendrocyte injury, oligodendrocytes (OLs) cultured in a Transwell system were analyzed after various treatments. Since Oligodendrocytes convert from a simple bipolar morphology to a complex branched morphology during differentiation [26], the morphological complexity of differentiating Oligodendrocytes processes was quantified using Sholl analysis (Fig. 8A, B), which revealed that OGD led to a significant decrease in the number of intersections, indicating inhibition of differentiation. Co-culture with untreated BV2 cells slightly mitigated this effect, but co-culture with LPS-IFN-γ-treated BV2 cells exacerbated the inhibition. Notably, co-culture with LPS-IFN-γ-treated CD36-KD BV2 cells resulted in more frequent intersections compared to BV2 cells (Fig. 8A-B). This suggests that CD36 knockdown can partially restore oligodendrocyte morphology during differentiation. Furthermore, co-culture with BV2 cells did not increase the number of MBP ^+^ multipolar cells. However, BV2 cells pretreated with LPS-IFN-γ significantly reduced the number of MBP ^+^ multipolar cells, which was reversed by CD36 knockdown (Fig. 8C). The expression levels of MBP were also reduced in OGD OLs co-cultured with LPS-IFN-γ-treated BV2 cells. As expected, CD36 knockdown in BV2 cells restored MBP expression levels in OGD OLs (Fig. 8D-E). These findings were further supported by cell viability assays and LDH release assays, which showed that CD36 knockdown mitigated the detrimental effect of LPS-IFN-γ-pretreated BV2 cells on OGD OLs (Fig. 8F–G).

## Discussion

In this study, we elucidate, for the first time, the ameliorative impact of CD36 receptor inhibition on WMI precipitated by TBI, along with its underlying cellular and molecular mechanisms. Subsequent to TBI, resting microglia undergo activation and polarization. Notably, a pronounced shift towards the pro-inflammatory phenotype exacerbates WMI. Our investigations in vivo and in vitro, for the first time, reveal that suppression of CD36 and its downstream Traf5-MAPK signaling pathway diminishes the prevalence of pro-inflammatory polarized microglia while augmenting anti-inflammatory polarization, thereby mitigating WMI post-TBI. These findings suggest that CD36 receptor inhibition may represent a viable therapeutic strategy to prevent the induction of deleterious pro-inflammatory microglia polarization following TBI.

The pathophysiology of traumatic brain injury (TBI) is characterized by a high level of complexity. Key mechanisms involved in secondary damage post-TBI, such as inflammatory responses, excitotoxicity, oxidative stress, and mitochondrial dysfunction, often intertwine, collectively impacting the brain [27–29]. Focusing on inflammation, microglia and macrophages, as primary defensive cells in cranial trauma, are rapidly recruited and activated at the injury site. This activation leads to the release of pro-inflammatory mediators, initiating an inflammatory response [30]. This response is intricately connected with other damage mechanisms. For example, mitochondrial dysfunction and oxidative stress can exacerbate inflammation [31, 32], and in some cases, inflammation can amplify excitotoxicity [33]. Recent studies have illuminated the remarkable plasticity of microglia/macrophages, which, upon activation, can adopt two principal phenotypes with distinct functional roles [34, 35]. The pro-inflammatory phenotype microglia secrete inflammatory cytokines, whereas the anti-inflammatory phenotype exhibit enhanced phagocytic capabilities and release a plethora of neurotrophic factors [36]. The pro-inflammatory phenotype is associated with exacerbation of neurological damage, while the anti-inflammatory phenotype is considered neuroprotective [25, 37]. Intriguingly, the dynamic balance between pro- and anti-inflammatory phenotype shifts over time following TBI and stroke, with anti-inflammatory cells predominating in the early phase post-injury, but a subsequent shift towards pro-inflammatory polarization becoming evident from day 7 onwards [38, 39]. This temporal pattern suggests an initial state of neural repair that gradually transitions to a state of sustained neuroinflammation.

Moreover, our findings indicate that CD36 expression peaks on day 7 post-injury, which, given the temporal pattern of microglia polarization [38, 39], suggests a robust association between CD36 and the activation of pro-inflammatory microglia. Interestingly, microglia are recognized as a specialized subset of macrophages [40]. CD36 receptors in macrophages were found to mediate inflammatory responses via mitochondrial oxidative stress or activation of the AMPK pathway [41, 42], and it was also revealed that LPS induces pro-inflammatory polarization of macrophages via CD36 in acute lung injury [43]. Collectively, these findings further support the link between CD36 and microglia polarization into pro-inflammatory phenotype. CD36 is implicated in a myriad of neuropathological processes, including cerebral ischemia [44, 45], neurovascular dysfunction [46], and atherosclerosis [47]. The CD36 antagonist SSO has been shown to mitigate stroke-induced neuroinflammation and confer neuroprotection in murine models of ischemic stroke [16]. Corroborating these findings, our study demonstrates that CD36 deletion attenuates myelin damage and oligodendrocyte loss following TBI. To assess the impact on neurological function post-WMI, we conducted a series of neurobehavioral tests to evaluate cognitive and motor functions in mice. Mice deficient in CD36 exhibited superior neurological recovery post-TBI compared to their wild-type counterparts. This is in alignment with Scott et al.‘s findings, which indicated enhanced neurological recovery in CD36 knockout mice following spinal cord injury [48]. CD36 is predominantly expressed in microglia and astrocytes within the brain [49], as confirmed by our experiments, and is known to modulate inflammatory responses and glial scarring [44, 45, 50, 51]. Furthermore, CD36 expression in vascular endothelial cells has been implicated in fatty acid transport [52] and the regulation of angiogenesis [13]. Notably, CD36-expressing macrophages can also infiltrate into the injured area and perform functions after spinal cord injury [48]. To elucidate the mechanisms by which CD36 inhibition ameliorates TBI-induced WMI, we performed transcriptome sequencing comparing CD36^(-/-)^ and wild-type mice post-TBI. The data suggest that modulation of microglial activation and neuroinflammation is a key mechanism by which CD36 inhibition exerts protective effects on white matter integrity. This substantiates the link between CD36 receptor activity and microglial activation. Through immunofluorescence, immunoblotting, and other experimental modalities, we have further confirmed that CD36 inhibition curtails the polarization of microglia towards the pro-inflammatory phenotype, thereby reducing myelin damage.

Subsequent transcriptome sequencing analysis of BV2 cells and molecular assays have corroborated the modulatory influence of the Traf5-MAPK (p38) axis on microglial polarization subsequent to TBI. The attenuation of the Traf5-MAPK axis was observed to inhibit the emergence of pro-inflammatory polarized microglia, thereby mitigating the WMI induced by TBI. Tumor necrosis factor receptor-associated factor 5 (Traf5) is an adaptor molecule associated with the tumor necrosis factor (TNF) receptor superfamily and the interleukin-1 receptor/Toll-like receptor superfamily, playing a pivotal role in the modulation of multiple signaling cascades [53]. Previous research has indicated that Traf5 upregulation occurs in glial cells and neurons post-spinal cord injury [54], and that a deficiency in Traf5 confers protection against cerebral ischemia-reperfusion injury by dampening inflammation and preserving the integrity of the blood-brain barrier (BBB) [53]. In the current study, the effects of LPS-IFN-γ in promoting pro-inflammatory polarization were significantly diminished following Traf5 inhibition via lentiviral transduction in BV2 cells. Correspondingly, mice administered with Traf5-shRNA exhibited a marked decrease in pro-inflammatory microglia phenotype on the seventh day post-TBI, relative to mice that underwent TBI alone. These findings lend further support to the anti-inflammatory properties of TRAF5 inhibition.

Mitogen-activated protein kinases (MAPKs), encompassing p38, ERK1/2, and JNK, belong to the serine-threonine protein kinase family and are implicated in the upregulation of pro-inflammatory cytokines [55, 56]. The MAPK signaling pathway has been reported to be significantly activated in conditions such as TBI [57], stroke [16], and spinal cord injury [58], and the inhibition of this pathway has been shown to provide neuroprotection in murine models of TBI [59, 60]. Our investigation into the role of p38 in the regulation of microglial activation, neuroinflammation, and white matter injury was facilitated by the use of a specific p-p38 inhibitor (SB242235). Aligning with the findings from previous studies, our results indicate that the suppression of p38 phosphorylation inhibits the pro-inflammatory polarization of microglia, thereby alleviating WMI induced by TBI. Collectively, these findings suggest that the inhibition of CD36 can prevent white matter injury by modulating microglial polarization through the Traf5-MAPK signaling pathway.

While our study pioneers in demonstrating that inhibiting CD36 can alleviate white matter injury post-TBI, it presents certain limitations. Primarily, the applicability of conclusions drawn from murine models to humans remains uncertain. Furthermore, despite extensive research on the CD36 receptor inhibitor SSO, its clinical safety and effectiveness remain unverified. Additionally, considering that pharmacological analgesia might interfere with the results of some experiments, no analgesic drugs were administered to the mice, which, however, may carry the inherent risk of introducing biases into the neurobehavioral test results. And the data collection and analysis in neurobehavioral tests rely on evaluators, who might overlook subtle yet relevant changes. Future research should, therefore, focus on expanding animal studies to gather substantial data, developing CD36-specific inhibitors, and conducting clinical trials to assess the safety and effectiveness of CD36 inhibitors, offering new therapeutic avenues for TBI patients.

## Conclusions

In summary, the findings of our investigation offer robust support for the premise that the inhibition of CD36 is instrumental in averting white matter injury. This protective effect is achieved by the modulation of microglial polarization, steering cells away from the pro-inflammatory phenotype via the downregulation of the Traf5-MAPK signaling pathway, as depicted in the graphical abstract. The data advocate for the targeting of CD36 expression in microglia as a viable therapeutic strategy to mitigate TBI-induced neuroinflammation and the ensuing white matter injury. This research enriches our understanding of the pathophysiological mechanisms underlying white matter injury post-TBI and introduces a potential therapeutic avenue for TBI clinical interventions.

### Electronic supplementary material

Below is the link to the electronic supplementary material.

Supplementary Material 1

Supplementary Material 2

## Data Availability

All data used during the current study available from the corresponding author on reasonable request.

## Abbreviations

Arg1: Arginase 1
bFGF: Basic fibroblast growth factor
BP: Biological processes
BSA: Bovine serum albumin
CC: Corpus callosum
CCI: Controlled cortical impact
CNS: Central nervous system
CNTF: Ciliary neurotrophic factor
DEGs: Differentially expressed genes
DMEM: Dulbecco’s modified eagle medium
GFAP: Glial fibrillary acidic protein
GSEA: Gene Set Enrichment Analysis
Iba-1: Ionized calcium-binding adapter molecule-1
IFN-γ: Interferon gamma
IL-4: Interleukin 4
iNOS: Inducible nitric oxide synthase
KD: Knockdown
KO: Knockout
LDH: Lactate dehydrogenase
LFB: Luxol fast blue
LPS: Lipopolysaccharides
MAPK: Mitogen-activated protein kinase
MBP: Myelin basic protein
NeuN: Neuron-specific nuclear protein
OE: Overexpress
OGD: Oxygen glucose deprivation
Olig2: Oligodendrocyte Lineage Transcription Factor 2
OLs: Oligodendrocytes
OPCs: Oligodendrocyte precursor cells
PDGF: Platelet-derived growth factor
p-p38: Phosphorylated p38
SMI-32: Neurofilament H (NF-H)
SSO: Sulfosuccinimidyl oleate
T3: Triiodothyronine
TBI: Traumatic brain injury
TNF: Tumor necrosis factor
Traf5: Tumor necrosis factor receptor-associated factor 5
WMI: White matter injury
