# Supplementary material for: CD36 deletion prevents white matter injury by modulating microglia polarization through the Traf5-MAPK signal pathway
Source: J Neuroinflammation. 2024 Jun 5;21:148. doi: 10.1186/s12974-024-03143-2 (PMC11155181; doi:10.1186/s12974-024-03143-2)
Supplement: Supplementary file 1 — Supplementary Material 1 [file 12974_2024_3143_MOESM1_ESM.docx]

| Substances | Sources |
| --- | --- |
| Dulbecco's Modified Eagle Medium (DMEM) | Gibco, USA |
| penicillin-streptomycin | Sigma, USA |
| fetal bovine serum | Gibco, USA |
| Trypsin | Sigma, USA |
| poly-D-lysine | Sigma, USA |
| DMEM/F12 | Gibco, USA |
| L-glutamine | Gibco, USA |
| sodium pyruvate | Gibco, USA |
| nonessential amino acids | Gibco, USA |
| penicillin | Sigma, USA |
| streptomycin | Sigma, USA |
| bovine serum albumin | Gibco, USA |
| human apo-transferrin | Invitrogen, USA |
| insulin | Gibco, USA |
| sodium selenite | Sigma, USA |
| D-biotin | Invitrogen, USA |
| hydrocortisone | Sigma, USA |
| platelet-derived growth factor (PDGF) | Gibco, USA |
| basic fibroblast growth factor (bFGF) | Gibco, USA |
| triiodothyronine (T3) | Sigma, USA |
| ciliary neurotrophic factor (CNTF) | Sigma, USA |

**Table. S1** Sources of material for cell culture
