# Supplementary material for: CD36 deletion prevents white matter injury by modulating microglia polarization through the Traf5-MAPK signal pathway
Source: J Neuroinflammation. 2024 Jun 5;21:148. doi: 10.1186/s12974-024-03143-2 (PMC11155181; doi:10.1186/s12974-024-03143-2)
Supplement: Supplementary file 2 — Supplementary Material 2 [file 12974_2024_3143_MOESM2_ESM.docx]

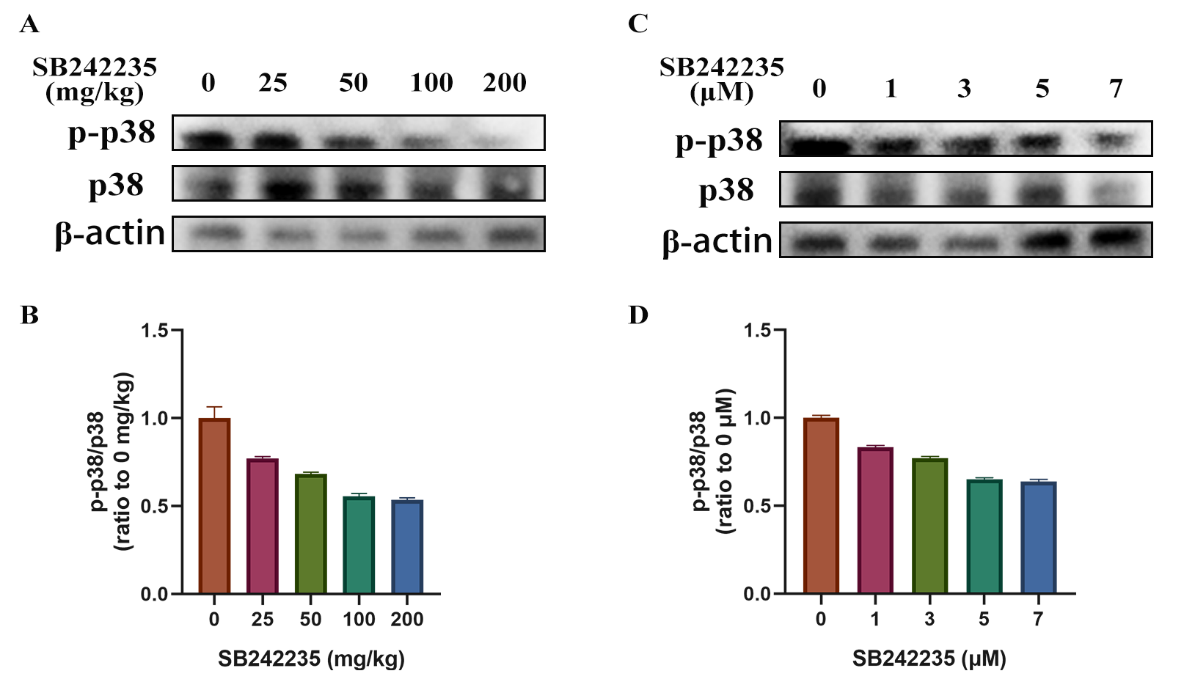


Supplementary figure 1. Dose-response study of SB242235 in vivo and in vitro. Assessment of the p38 Phosphorylation degree at different concentrations of SB242235 by western blot and quantification in vivo (A, B) and in vitro (C, D).


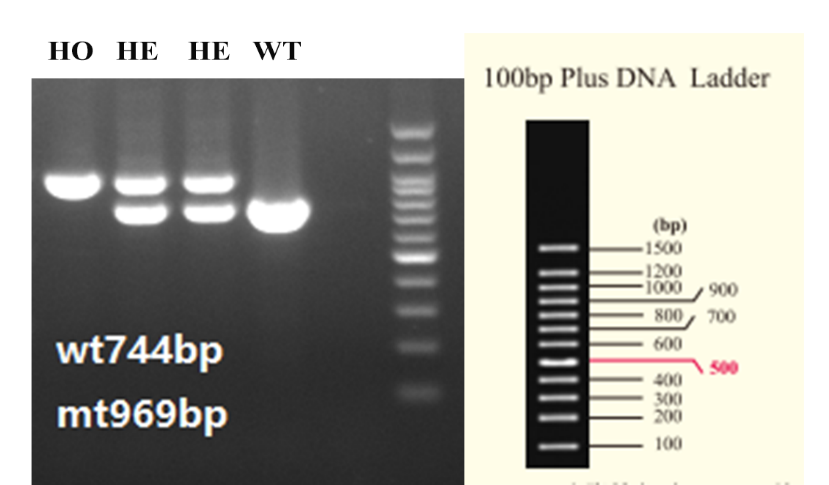


Supplementary figure 2. Identification of Knockout Mice by DNA Agarose gel Electrophoresis


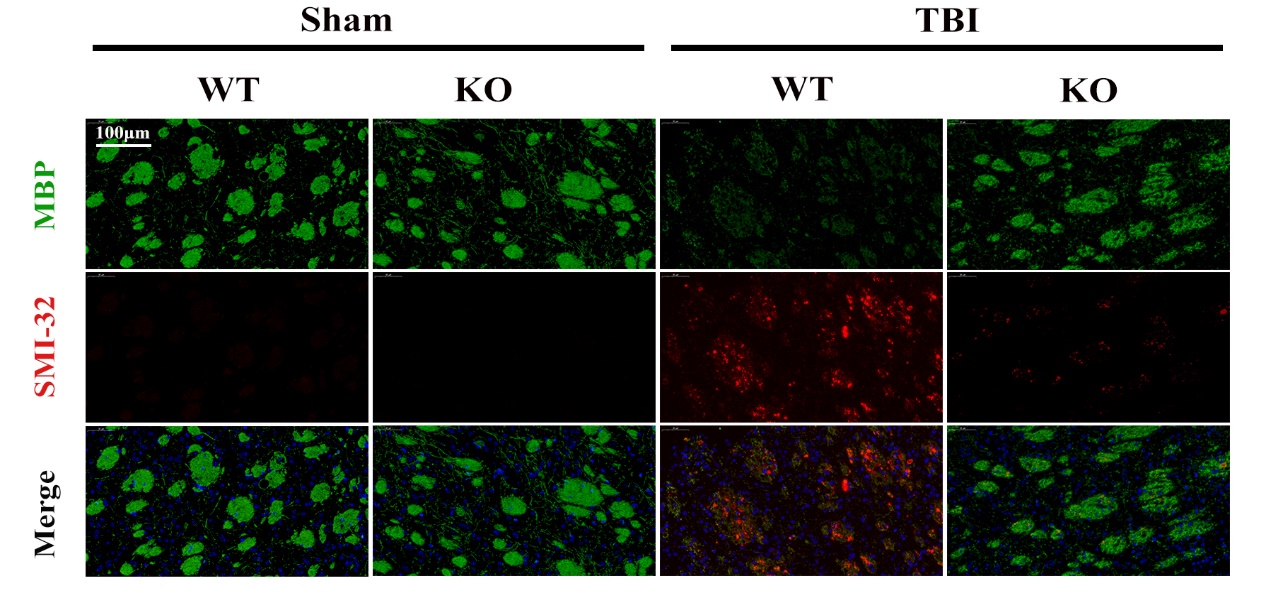


Supplementary figure 3. Dual immunofluorescence staining for dephosphorylated neurofilament protein (SMI-32, red) and myelin basic protein (MBP, green) in the ipsilateral striatum at day 7 post-TBI, with DAPI staining nuclei (blue). Scale bar: 100 μm.


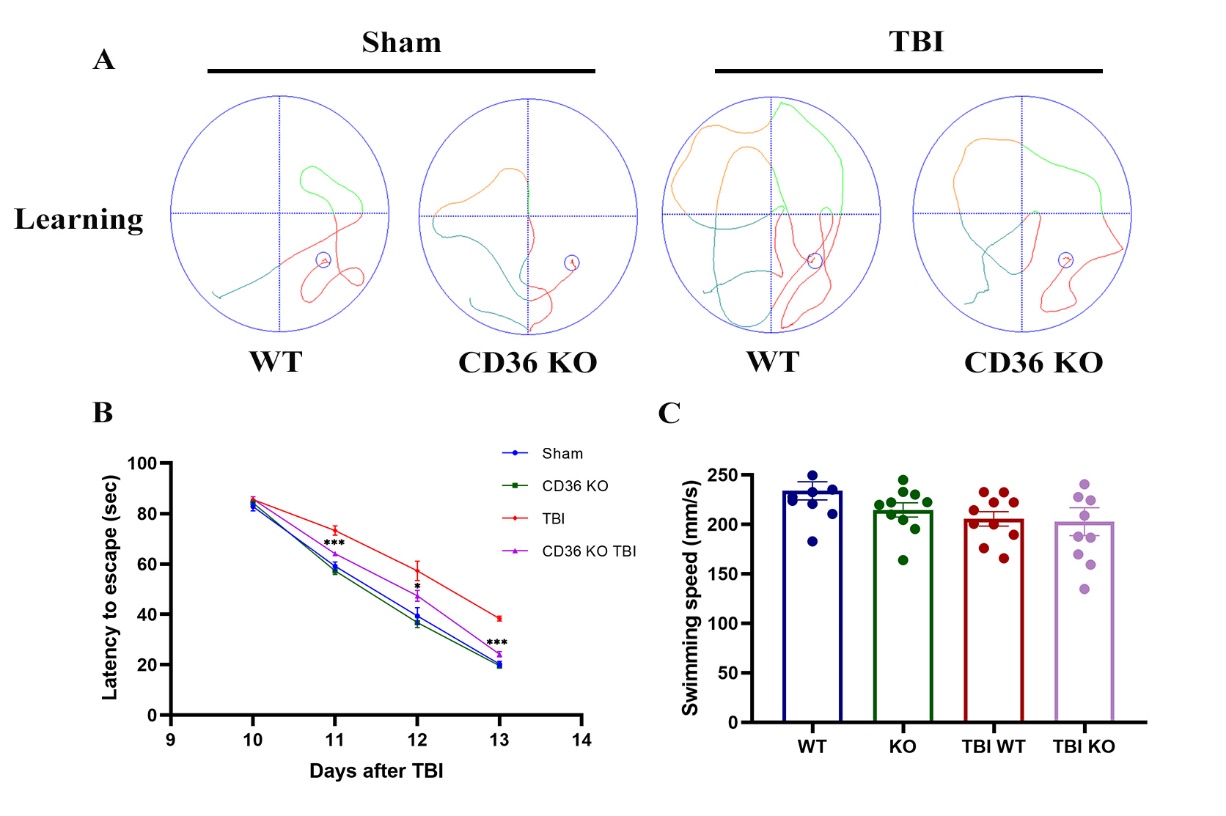


Supplementary figure 4. The training phase of Morris water maze tests. (A) Swim paths depicted during the trial; (B) time spent on escape; (C) swim speed of each group. Data represent mean ± SEM for 10 mice per group. *P < 0.05, **P < 0.01, ***P < 0.001.


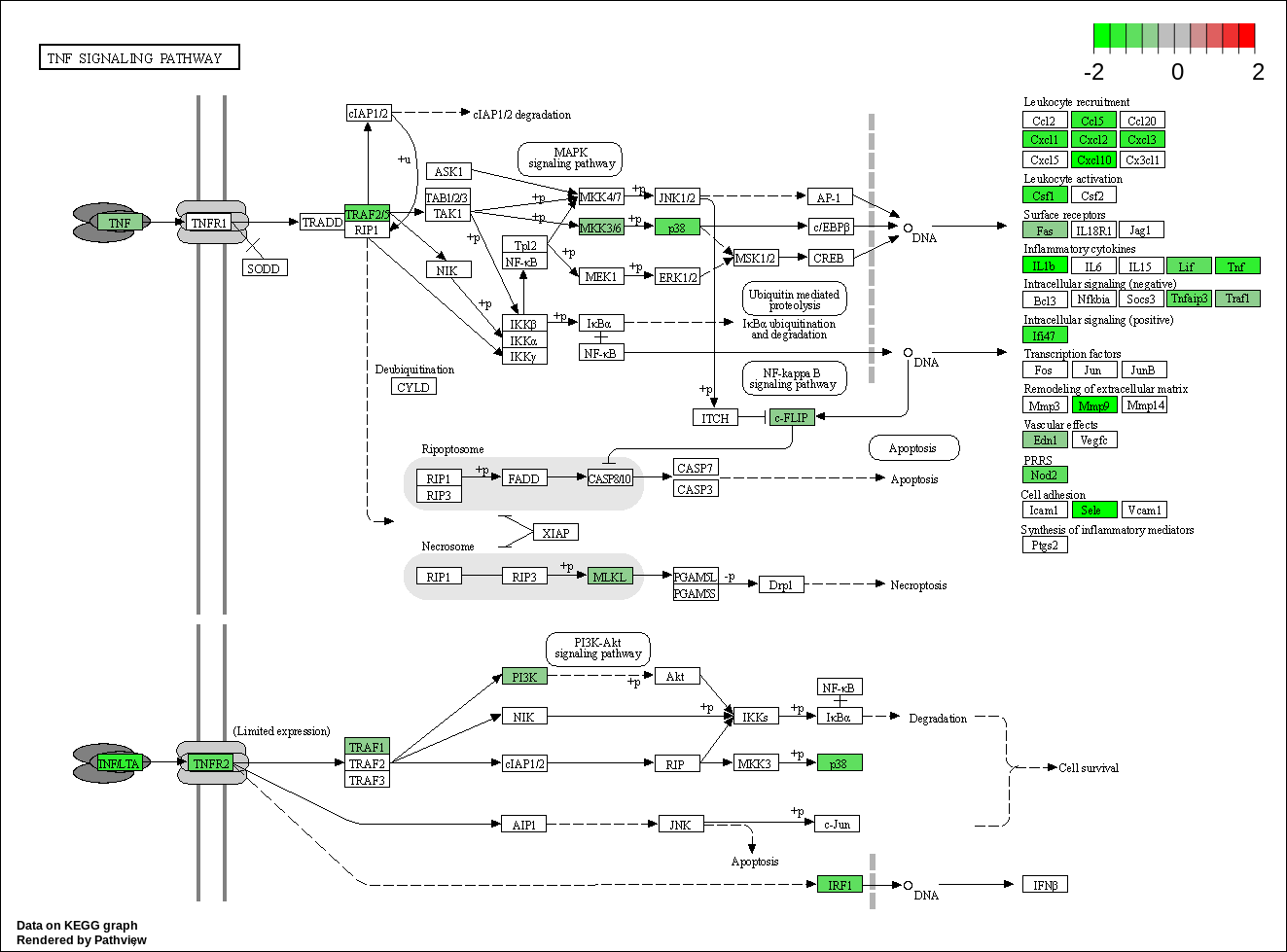


Supplementary figure 5. KEGG enrichment analysis of TNF signaling pathway.
